# Rare monomorphic epithelial intestinal T-cell lymphoma of the stomach with a giant gastric perforation rescued by liver-covering sutures followed by a total gastrectomy and lateral hepatectomy: a case report

**DOI:** 10.1186/s40792-022-01381-1

**Published:** 2022-02-07

**Authors:** Keiji Muramoto, Sachiko Kaida, Toru Miyake, Rie Nishimura, Katsuyuki Kito, Masanori Shiohara, Ryoji Kushima, Tomoharu Shimizu, Masaji Tani

**Affiliations:** 1 Department of Surgery, Shiga University of Medical Science, Seta, Tsukinowa-cho, Otsu, Shiga 520-2192 Japan; 2 Department of Gastroenterology and Hematology, Shiga University of Medical Science, Seta, Tsukinowa-cho, Otsu, Shiga 520-2192 Japan; 3 Department of Clinical Laboratory Medicine and Diagnostic Pathology, Shiga University of Medical Science, Seta, Tsukinowa-cho, Otsu, Shiga 520-2192 Japan; 4 Medical Safety Section, Shiga University of Medical Science Hospital, Seta, Tsukinowa-cho, Otsu, Shiga 520-2192 Japan

**Keywords:** Monomorphic epitheliotropic intestinal T-cell lymphoma, Gastric lymphoma, Perforation, Hepatectomy

## Abstract

**Background:**

Monomorphic epitheliotropic intestinal T-cell lymphoma (MEITL), a type of peripheral T-cell lymphoma, rarely involves the stomach as the primary organ. Advanced MEITL, for which there is currently no established treatment, causes gastrointestinal perforations and is characterized by a poor response to chemotherapy.

**Case presentation:**

A 69-year-old man had undergone chemotherapy for MEITL of the whole stomach. He subsequently developed acute abdominal pain, and computed tomography revealed a giant perforation in the anterior gastric wall adjacent to the lateral segment of the liver. The perforation was rescued through closure with liver-covering sutures. Thereafter, a total gastrectomy and a left hepatectomy were performed and he recovered enough to tolerate oral intake. However, despite ongoing chemotherapy, the patient died 83 days after the gastric perforation (10 months after being diagnosed with the lymphoma) owing to rapid progression of the MEITL.

**Conclusion:**

In the rare case of a giant gastric perforation after chemotherapy for gastric MEITL, rescue is possible through liver-covering sutures followed by a total gastrectomy and lateral hepatectomy.

## Background

Enteropathy-associated T-cell lymphoma (EATL) is classified as a rare type of peripheral T-cell lymphoma of the gastrointestinal tract, accounting for less than 1% of non-Hodgkin lymphoma [1]. In the first place, EATL shows specific findings of villi atrophy for a pathological feature [2]. EATL occurs most frequently in the small intestine, and rarely occurs in the stomach at 8% [3].

EATL can be classified into two different types according to the 2016 World Health Organization classification of lymphoid neoplasms [4]. Type 1 EATL, also known as classic EATL, develops against the background of celiac disease and, therefore, has a high incidence in Europe and North America. It is often the cause of death in patients with refractory celiac disease. Type 2 EATL, which is not associated with celiac disease, is named monomorphic epitheliotropic intestinal T-cell lymphoma (MEITL) and is characterized by the infiltration of monomorphic lymphoma cells into the ductal epithelium. Both EATL types have different immunohistochemical characteristics [1]. Classic EATL and MEITL often form multiple ulcers and cause intestinal perforation, and it is considered to have a clinically aggressive course. We report a rare case of gastric MEITL perforation after chemotherapy.

## Case presentation

**Fig. 1:**
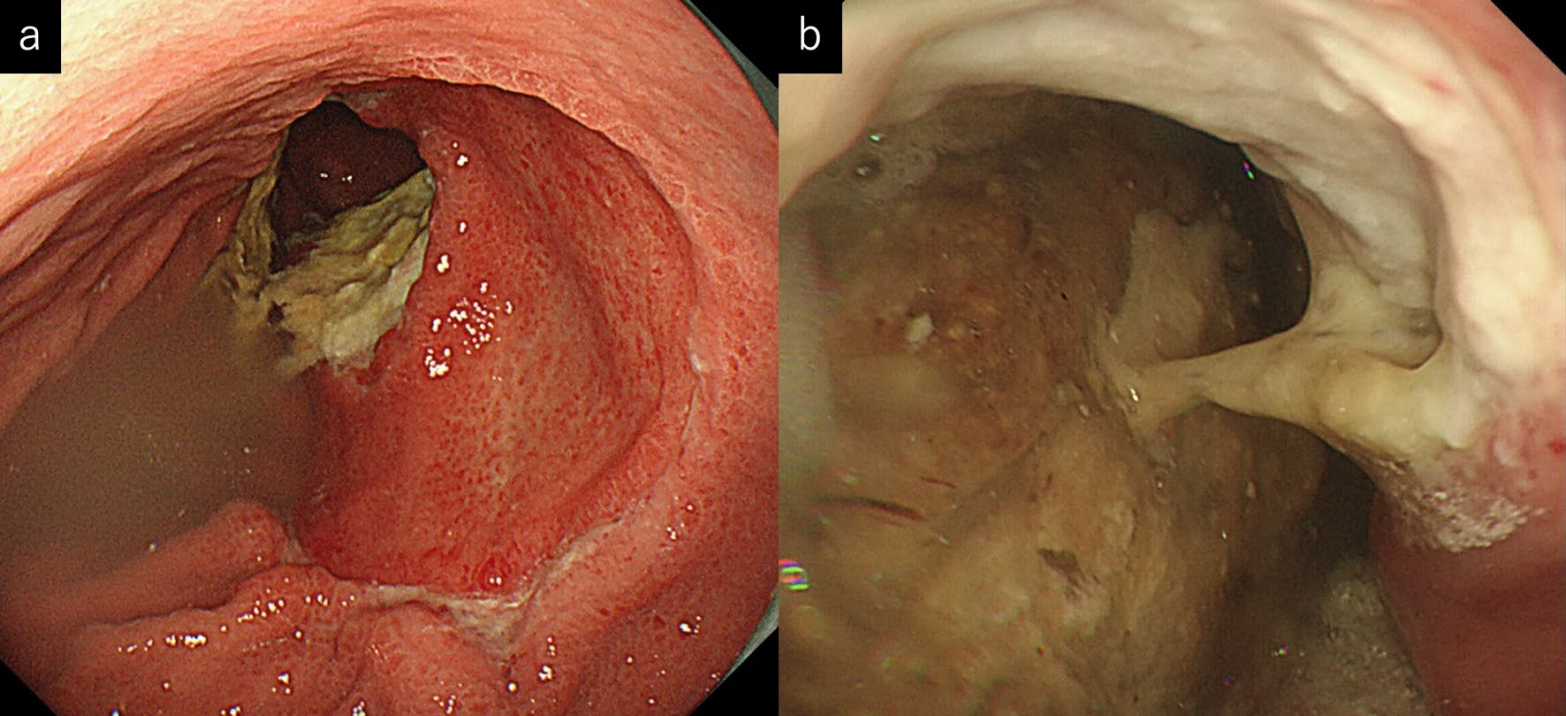
**a** Upper gastrointestinal endoscopy showed irregular ulcers on the small side of the gastric body. **b** After 8 courses of CHOP (cyclophosphamide 750 mg/m^2^, doxorubicin 50 mg/m^2^, vincristine 1.4 mg/m^2^, prednisolone 100 mg) therapy, ulcers tended to increase

A 69-year-old man presented with epigastric pain. An upper gastrointestinal endoscopy scan revealed irregular ulcers in the lesser curvature of the stomach (Fig. 1). Hematoxylin and eosin staining of the biopsy sample from the stomach lesions showed intraepithelial infiltration by atypical lymphocytes. Immunohistochemical staining for T-cell surface markers revealed a CD3(+), CD4(–), CD5(–), and CD7(+) immunophenotype.

The patient was diagnosed with malignant primary gastric lymphoma, because (1) the ^18^F-fluorodeoxyglucose (FDG) positron emission tomography–computed tomography can revealed accumulation of FDG at the stomach wall only, and (2) no abnormal lesion was evident from the colonoscopy and small intestinal endoscopy scans.

**Fig. 2:**
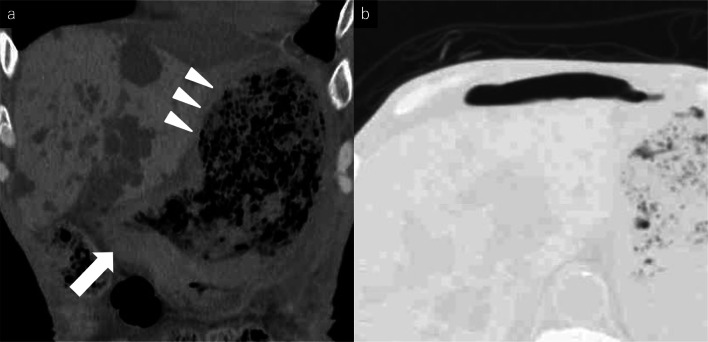
**a** Abdominal CT showed pyloric stenosis (arrow) and extensive gastric wall defects on the lesser curvature of the stomach (arrowhead). **b** A large amount of free air was observed around the liver

**Fig. 3:**
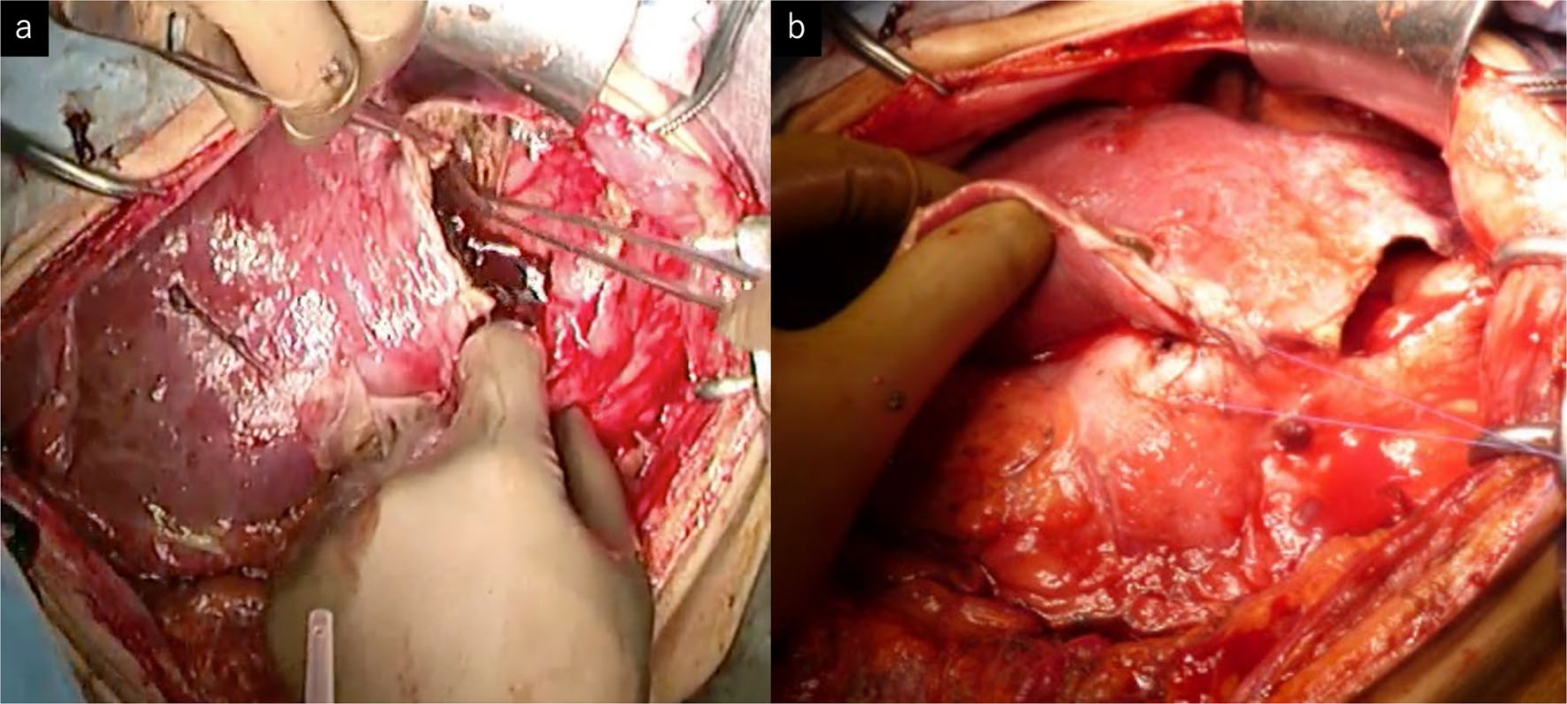
**a** Anterior gastric wall was thinned and adhered to the lateral hepatic segment. The perforated part was 8 cm in diameter. **b** Entire stomach wall and the margin of the outer hepatic segment were sutured directly to close the perforation

Eight courses of first-line CHOP therapy (cyclophosphamide 750 mg/m^2^, doxorubicin 50 mg/m^2^, vincristine 1.4 mg/m^2^, and prednisolone 100 mg/bid) were administered to the patient. Consequently, his gastric ulcers tended to increase (Fig. 1), and his CT scans revealed that the tumor diameter in the ulcer area had increased by 61% (from 21 to 34 mm). The soluble interleukin-2 receptor level had also increased from 682 U/mL pre-chemotherapy to 1170 U/mL after the eight courses of chemotherapy. Because of the poor control of the tumor, we switched to pralatrexate (30 mg/m^2^) as the second-line treatment. However, the patient developed acute abdominal pain on the fourth day following the last cytotoxic drug administration. Physical examinations revealed board-like stiffness of the abdomen, a low blood pressure of 88/52 mmHg, and a pulse of 88 beats/min. Laboratory examinations revealed a hemoglobin count of 6.4 g/dL, a white blood cell count of 6400/μL, and a C-reactive protein level of 5.14 mg/dL. The abdominal CT scan revealed pyloric stenosis, a large amount of residual fluid in the stomach, and extensive gastric wall defects on the side of the lesser curvature of the gastric body (Fig. 2). The patient was diagnosed with peritonitis due to a hemorrhagic gastric perforation, and an emergency laparotomy was performed. Necrosis of the gastric mucosa was observed around the giant perforation, and the tumor had invaded the lateral segment of the liver. Because it was difficult to close the perforation site with a simple suture, the perforated part was emergently closed by suturing the lateral segment of the liver and the stomach wall together (Fig. 3). Although the patient needed 11 days of recovery in the intensive care unit after surgery, his general condition improved. With regard to the therapeutic strategy, many physicians on the cancer board had concluded that a tumor resection should be done for allowing oral intake; therefore, a total gastrectomy and a partial hepatectomy were performed 39 days after the first emergency operation. The patient was able to take a meal on the fourth day after the total gastrectomy.

**Fig. 4:**
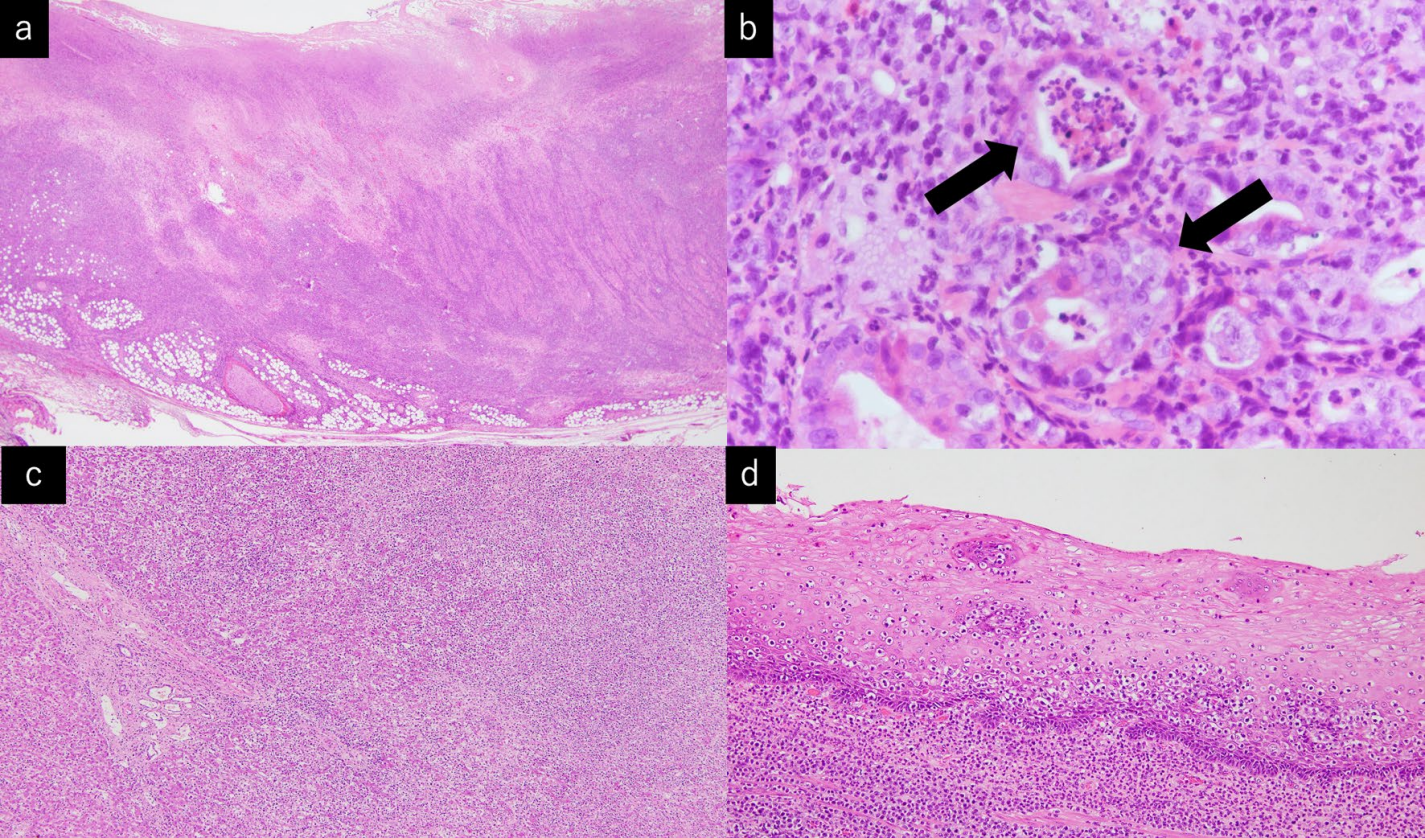
**a** Histopathological findings showed deformation of the gastric gland structure. **b** infiltration of atypical cells into the epithelium and destruction. **c** Atypical cell infiltration into the liver. **d** Atypical cell infiltration into the esophagus

**Fig. 5:**
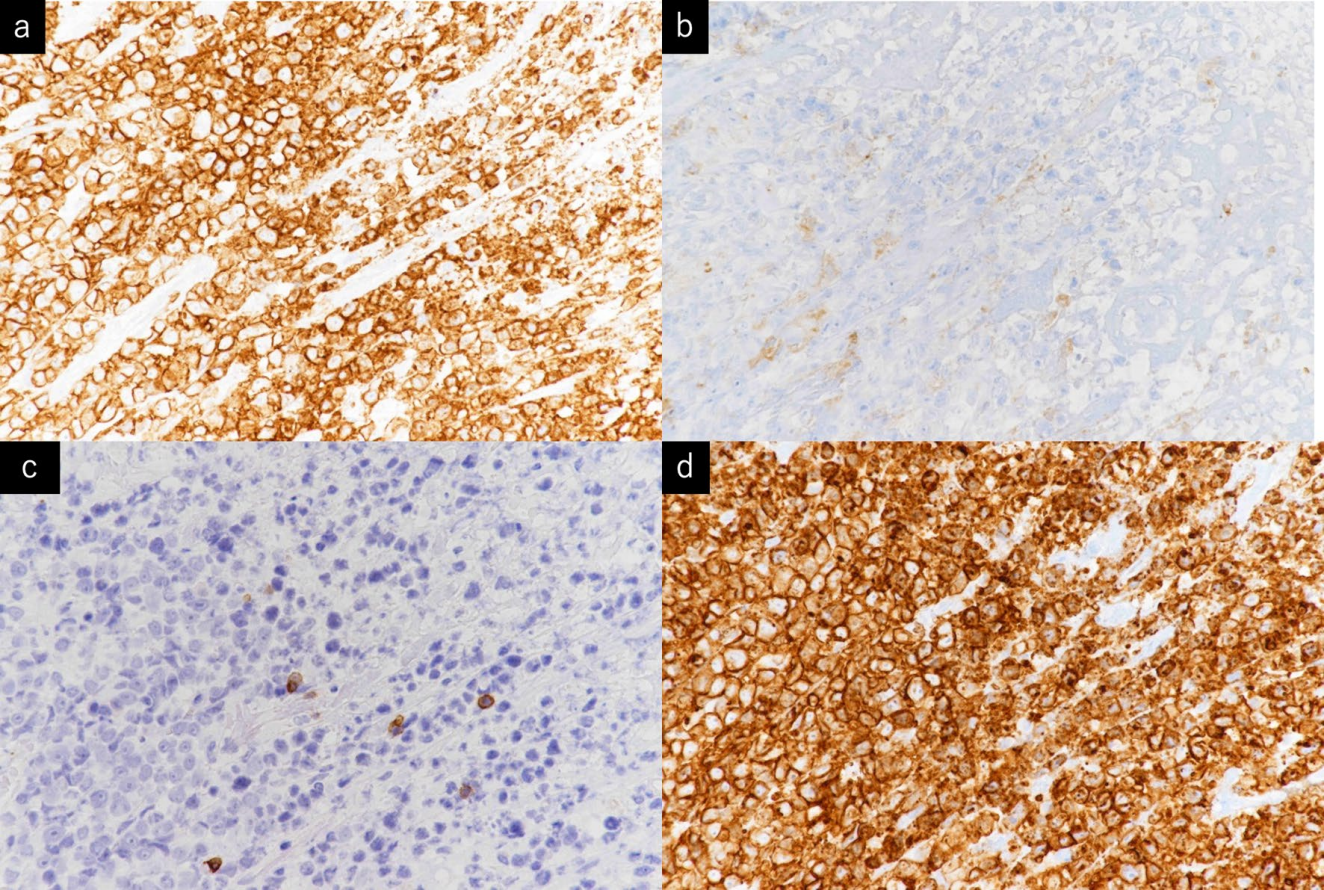
Immunohistochemical staining showed CD3 (+), CD4 (−), CD5 (−), CD7 (+). The tumor cells were thought to be derived from intestinal intraepithelial T cells. **a** CD3 (+). **b** CD4 (−) **c** CD5 (−) **d** CD7 (+)

Histopathological examination of the resected stomach and liver revealed atypical cell infiltration into the epithelium, destruction and deformation of the gastric wall, and infiltration into the liver and esophageal mucosa (Fig. 4). The microscopy investigation revealed that there were no tumor cells at the surgical margins. Upon immunohistochemical staining, T cells showed a CD3(+), CD4(–), CD5(–), and CD7(+) immunophenotype (Fig. 5) and gene rearrangement of the T-cell receptor gamma-chain.

**Fig. 6:**
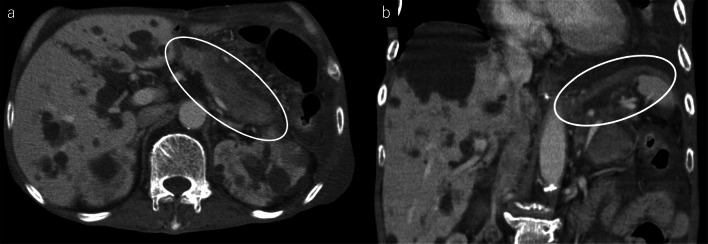
**a** CT after total gastrectomy shows a low absorption region in the pancreas tail which is enlarged. **b** Left diaphragm was also thickened, and tumor infiltration was suspected in these lesions

However, at 30 days after the secondary surgery, a CT scan revealed recurrent tumors in the pancreatic tail and the left diaphragm (Fig. 6). Therefore, IVE therapy (ifosfamide 3000 mg/m^2^, epirubicin 50 mg/m^2^, and etoposide 200 mg/m^2^) was performed as the third line of therapy but was unfortunately ineffective, and the patient died of respiratory deterioration due to pneumonia and pleural effusion on day 44 (83 days after discovery of the gastric perforation). The survival period was 10 months from the diagnosis of lymphoma.

The diagnosis of a primary malignant lymphoma of the digestive tract is based on the following diagnostic criteria of Dawson et al. [5]: (1) gastrointestinal lesions are predominant, and metastasis is only in the regional lymph nodes; (2) there is no swelling of the superficial lymph nodes; (3) there is no mediastinal lymphadenopathy on simple chest X-rays; (4) hematological examination of the peripheral blood shows no whitening; and (5) there are no tumors in the liver and spleen. Primary gastric lymphomas derived from T cells are extremely rare. EATL and MEITL accounts for less than 1% of all non-Hodgkin’s lymphomas [6]. Although primary gastrointestinal T-cell lymphomas occur predominantly in the small intestine, 8% of the tumors originate in the stomach [3]. In a PubMed search, we found only two patients with classic EATL or MEITL involving the stomach. One of these patients was a 73-year-old man who was diagnosed with classic EATL after a subtotal gastrectomy for a gastric perforation. The patient lived without any tumor recurrence or metastases within 3 months of follow-up [7]. The other patient, a 65-year-old male diagnosed with MEITL from gastrointestinal bleeding, had died 13 months after diagnosis [8].

Histologically, MEITL is characterized by the infiltration of atypical monoclonal lymphoid cells into the ductal epithelium, and unlike classic EATL, there is no infiltration of reactive inflammatory cells into the surroundings. Lymphoma cells have a CD3(+), CD4(–), CD5(–), CD7(+), and CD103(+) immunophenotype with T-cell receptor beta-chain(–) expression and contain cytotoxic granule proteins [8]. In our patient, the tumor cells were considered to be derived from T cells in the intestinal epithelium, because they presented a CD3(+), CD5(–), CD7(+), and CD4(–) immunophenotype and had a recognized gene rearrangement of the T-cell receptor gamma-chain, resulting in the T-cell lymphoma diagnosis. On the basis of these immunohistochemical findings, the possibility of a primary lymphoma of the gastrointestinal tract, and the lack of a background of celiac disease, we diagnosed the patient as having MEITL. PTL is classified as a lymphoma of intermediate-grade aggressiveness. Specifically, MEITL and classic EATL proliferate around the gastrointestinal tract wall and often cause gastrointestinal perforations. According to Sasaki et al. [9], the median survival time was 8 months for patients with a single perforation and 2.35 months for those with multiple perforations.

Our patient was diagnosed with MEITL and had developed a gastric perforation while undergoing chemotherapy, whereupon emergency surgery was performed to cover the lateral segment of the liver and suture it to the defect of the stomach wall. After the improvement of his general condition, a total gastrectomy and a lateral hepatic segmentectomy were performed. Postoperatively, the patient was able to tolerate oral intake and received chemotherapy for the lymphoma.

The tumor in our patient was localized to the stomach and was classified as Stage I according to the gastrointestinal Lugano classification (1994) [10]. Based on the international prognostic index [11], which is a prognostic indicator for patients with an aggressive lymphoma, the tumor presented a low to intermediate risk (age 61 years and older, high lactate dehydrogenase). The median survival time following CHOP-based chemotherapy, which is commonly administrated for the treatment of malignant lymphomas, is reportedly 7.5 months [12, 13]. Treatment is often not completed owing to adverse events or tumor progression. Although complete remission has been achieved in 35–40% of patients who completed treatment, relapse frequently occurs [14]. There are no established standard therapies for MEITL and classic EATL [15]. The median remission duration for both EATL types is only 6 months, and the 5-year survival rates are reported to be 9–22% [16]. In malignant lymphoma of the gastrointestinal tract, surgery is performed when tumor obstruction, hemorrhage or perforation has occurred or is expected to occur. It has been reported that the prognosis when the tumor can be completely resected is better than that when there is a residual lesion [17]. However, it has been reported that perforation or hemorrhage during chemotherapy for gastric malignant lymphoma is low frequency (less than 5%) [18], and there is a risk that gastrectomy may delay the introduction of chemotherapy, and the decreased functional prognosis due to postoperative complications and post-gastrectomy syndrome (dyspepsia and dumping syndrome) must be considered. In this case, prophylactic gastrectomy may have been considered before 2nd line chemotherapy in view of the gastrointestinal perforation and peritoneal dissemination that occurred. The timing of the surgery is a limitation of this presentation, because the stomach is an organ with a thicker muscular layer than the small intestine and early perforation is difficult to predict. In the future, the early diagnosis of MEITL and the establishment of standard treatments are still necessary to improve patient outcomes.

## Conclusion

We experienced an extremely rare case of primary gastrointestinal tract-associated T-cell lymphoma. It was possible to rescue the oncological emergency of a giant gastric perforation by suturing the lateral segment of the liver and the stomach wall together.

## Data Availability

All data analyzed during this study are included in this article.

## Abbreviations

MEITL: Monomorphic epitheliotropic intestinal T-cell lymphoma
EATL: Enteropathy-associated T-cell lymphoma
FDG: ^18^F-Fluorodeoxyglucose
IVE: Chemotherapy with ifosfamide, epirubicin and etoposide cisplatin
PET-CT: Positron emission tomography–computed tomography
CHOP: Chemotherapy with cyclophosphamide, doxorubicin, vincristine and prednisolone
IVE: Chemotherapy with ifosfamide, epirubicin and etoposide cisplatin
PTL: Peripheral T-cell lymphoma
IPI: International prognostic index
LDH: Lactate dehydrogenase
